# Simvastatin Improves Microcirculatory Function in Nonalcoholic Fatty Liver Disease and Downregulates Oxidative and ALE-RAGE Stress

**DOI:** 10.3390/nu14030716

**Published:** 2022-02-08

**Authors:** Evelyn Nunes Goulart da Silva Pereira, Beatriz Peres de Araujo, Karine Lino Rodrigues, Raquel Rangel Silvares, Carolina Souza Machado Martins, Edgar Eduardo Ilaquita Flores, Caroline Fernandes-Santos, Anissa Daliry

**Affiliations:** 1 Laboratory of Cardiovascular Investigation, Oswaldo Cruz Institute, Oswaldo Cruz Foundation, Pavilhão Ozório de Almeida Av. Brasil, 4365 (Room 14), Manguinhos, Rio de Janeiro 21040-900, RJ, Brazil; evyspereira@gmail.com (E.N.G.d.S.P.); bperes30@gmail.com (B.P.d.A.); karine_lino@hotmail.com (K.L.R.); raquelran@gmail.com (R.R.S.); carol.souzamachado@gmail.com (C.S.M.M.); edgar.microbiologo@gmail.com (E.E.I.F.); cf_santos@id.uff.br (C.F.-S.); 2 Department of Basic Sciences, Federal Fluminense University, Dr. Silvio Henrique Braune Street, 22, Nova Friburgo 28625-650, RJ, Brazil

**Keywords:** nonalcoholic fatty liver disease, simvastatin, microcirculation, liver, adipose tissue

## Abstract

Increased reactive oxidative stress, lipid peroxidation, inflammation, and fibrosis, which contribute to tissue damage and development and progression of nonalcoholic liver disease (NAFLD), play important roles in microcirculatory disorders. We investigated the effect of the modulatory properties of simvastatin (SV) on the liver and adipose tissue microcirculation as well as metabolic and oxidative stress parameters, including the advanced lipoxidation end product–receptors of advanced glycation end products (ALE-RAGE) pathway. SV was administered to an NAFLD model constructed using a high-fat–high-carbohydrate diet (HFHC). HFHC caused metabolic changes indicative of nonalcoholic steatohepatitis; treatment with SV protected the mice from developing NAFLD. SV prevented microcirculatory dysfunction in HFHC-fed mice, as evidenced by decreased leukocyte recruitment to hepatic and fat microcirculation, decreased hepatic stellate cell activation, and improved hepatic capillary network architecture and density. SV restored basal microvascular blood flow in the liver and adipose tissue and restored the endothelium-dependent vasodilatory response of adipose tissue to acetylcholine. SV treatment restored antioxidant enzyme activity and decreased lipid peroxidation, ALE-RAGE pathway activation, steatosis, fibrosis, and inflammatory parameters. Thus, SV may improve microcirculatory function in NAFLD by downregulating oxidative and ALE-RAGE stress and improving steatosis, fibrosis, and inflammatory parameters.

## 1. Introduction

Nonalcoholic fatty liver disease (NAFLD) is the main cause of liver dysfunction worldwide, affecting 20–30% of the adult population [1,2,3,4]. NAFLD is characterized by the accumulation of >5% fat in the liver in the absence of other chronic liver diseases and alcohol abuse [4,5]. NAFLD ranges from simple steatosis to nonalcoholic steatohepatitis (NASH), and is characterized by hepatic lesions, inflammation, and fibrosis [6,7]. NAFLD increases the risk of terminal liver disease, liver cirrhosis, and hepatocellular carcinoma [8,9]. Recently, an international expert panel proposed the term metabolic dysfunction-associated fatty liver disease “MAFLD” as a new terminology that more accurately reflects the pathogenesis of fatty liver disease [10]. MAFLD diagnosis includes evidence of hepatic steatosis in addition to at least one of the other three criteria: Overweight/obesity, presence of type 2 diabetes mellitus, or evidence of metabolic dysregulation [11]. A growing percentage of patients with NAFLD require liver transplantation. NASH is currently the second most prevalent cause of liver transplantation and may replace hepatitis as the leading cause of liver transplantation in the future as more hepatitis C virus (HCV)-infected patients are treated with highly curative antiviral regimens [12,13]. The etiology of NAFLD is multifactorial, wherein several parallel factors, including insulin resistance and abnormal lipid metabolism, synergistically contribute to the progression of NAFLD from benign steatosis to NASH [14,15].

While NAFLD is common, currently there are no approved pharmacological interventions for its treatment. Thus, liver transplantation remains the only effective therapy for several types of liver disease [16], justifying the need for new therapeutic alternatives or drug repositioning to minimize liver damage resulting from NAFLD. Studies conducted by us and others have indicated that disorders in hepatic microcirculation may play a critical role in the progression of NAFLD [16,17,18,19,20] and that the degree of steatosis is inversely related to hepatic microcirculatory blood flow. The decrease in hepatic blood flow may contribute to tissue damage via increased reactive oxidative stress (ROS) generation and lipid peroxidation [21,22]. Thus, the need for strategies that target vascular homeostasis in NAFLD is clinically relevant.

Statins competitively inhibit 3-hydroxy-3-methyl-glutaryl-CoA reductase (HMG-CoA), which limits endogenous biosynthesis of cholesterol and isoprenoids in the mevalonate pathway. This leads to a decrease in the levels of plasma cholesterol and lipoproteins as well as cholesterol synthesis in the liver, which increases the number of LDL receptors on the surface of hepatocytes, with a consequent rise in LDL absorption and catabolism [23]. Current clinical guidelines support the use of statins, such as simvastatin, for the treatment of dyslipidemia and for the secondary prevention of myocardial infarction in patients with NAFLD [24]. Statins reportedly induce a protective action against post-ischemic damage to the liver, heart, and brain [25,26,27] and enhance microcirculatory function [28,29,30]. However, studies on the pleiotropic and mechanistic effects exerted by statins on liver disease are scant, with most studies being based on the hypolipidemic action of these compounds [23]. 

In the present study, we used a mouse NAFLD model induced by a high-fat–high-carbohydrate (HFHC) diet. We investigated the mechanisms underlying the protective effects of simvastatin (SV) by evaluating the impact of SV treatment on hepatic and adipose tissue microcirculation, leukocyte recruitment, microcirculatory blood flow, sinusoid diameter, hepatic stellate cell (HSC) activation, and endothelial function. Moreover, we explored the modulatory effects exerted by SV on metabolic and oxidative stress parameters, including the advanced lipoxidation end product–receptors of advanced glycation end products (ALE-RAGE) pathway.

## 2. Materials and Methods

### 2.1. Animals and Experimental Protocol

**Figure 1:**
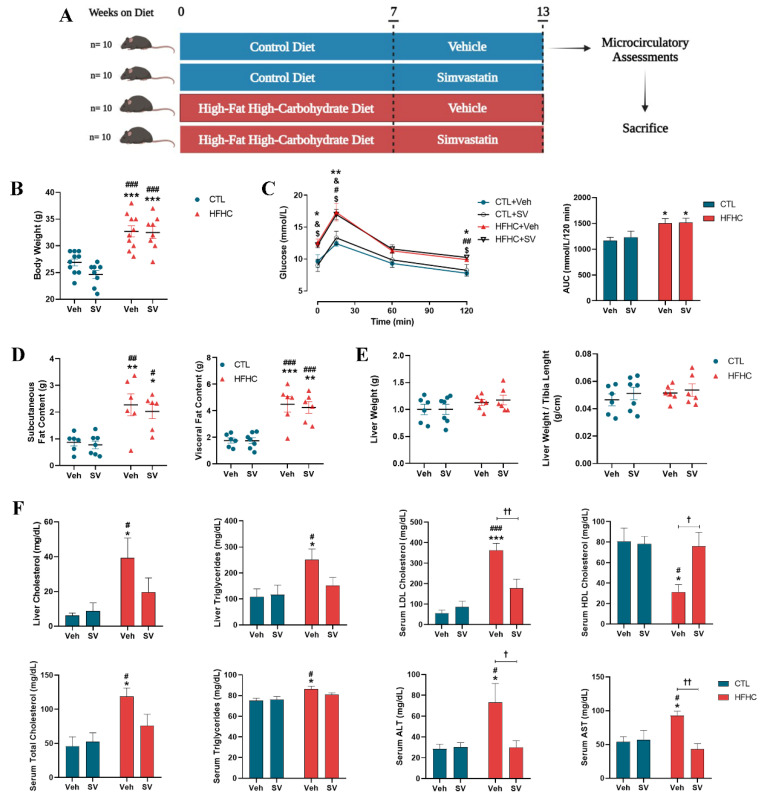
SV protects against nonalcoholic fatty liver disease (NAFLD)-induced metabolic changes. Schematic representation of the experimental protocol used to assess the effect of SV on NAFLD (**A**). Hemodynamic and metabolic parameters of mice fed control diet (CTL+Veh), control diet plus SV (CTL+SV), high-fat–high-carbohydrate (HFHC) diet (HFHC+Veh), or HFHC diet plus Simvastatin treatment (HFHC+SV) are show as: Body weight (**B**), fat content (**D**), liver weight (**E**) and biochemical parameters (**F**). * *p* < 0.05 vs. CTL+Veh; ** *p* < 0.01 vs. CTL+Veh; *** *p* < 0.001 vs. CTL+Veh; ^#^ *p* < 0.05 CTL+SV; ^##^ *p* < 0.01 CTL+SV; ^###^ *p* < 0.001 vs. CTL+SV; ^†^ *p* < 0.05 HFHC+SV; ^††^ *p* < 0.01 HFHC+SV. Oral glucose tolerance test (OGTT) is demonstrated in (**C**). Area under the curve (AUC) of glucose was calculated using the trapezoidal rule (**C**). * *p* < 0.05 CTL+Veh vs. HFHC+Veh; ** *p* < 0.01 CTL+Veh vs. HFHC+Veh; ^#^ *p* < 0.05 CTL+Veh vs. HFHC+SV; ^##^ *p* < 0.01 CTL+Veh vs. HFHC+SV; ^&^ *p* < 0.05 CTL+SV vs. HFHC+Veh; ^$^ *p* < 0.05 CTL+SV vs. HFHC+SV.

Forty 4-week-old male C57BL/6 mice were housed in polypropylene cages kept in a room under conditions of a 12 h light/dark cycle and a constant temperature of 22 ± 1 °C, with access to water and food ad libitum. NAFLD was induced using a high-fat diet (60% of calories from fat; D12492, Research Diets, New Brunswick, NJ, USA) in conjunction with a high-carbohydrate drink containing 45 kg/m^3^ carbohydrate (55% fructose and 45% sucrose) for 13 weeks (*n* = 20). The control (CTL) group (*n* = 20) without NAFLD were fed a control purified mouse diet (10% of calories from fat; D12450J, Research Diets, New Brunswick, NJ, USA) and water for 13 weeks. At week 7, the CTL and NAFLD groups were divided into two groups: (i) Untreated animals receiving vehicle between weeks 7 and 13 (CTL+Veh; *n* = 10 and HFHC+Veh; *n* = 10); and (ii) treated with SV (20 mg/kg/day) between weeks 7 and 13 (CTL+SV; *n* = 10 and HFHC+SV; *n* = 10). The 20 mg/kg SV dose used has been previously reported [31,32,33,34] and showed no adverse effects (did not cause mice mortality or hepatic toxicity evidenced by transaminase evaluation). At the end of the protocol, ketamine (100 mg/kg) and xylazine (10 mg/kg) were used to anesthetize the animals, following which blood, liver, subcutaneous and visceral (abdominal, retroperitoneal and epididymal grouped) adipose tissues were collected and weighed. Plasma aliquots and tissues were stored at −80 °C for further analysis (Figure 1). All animal experiments were approved by the Animal Welfare Committee of the Oswaldo Cruz Foundation (license L-019/2016).

### 2.2. Biochemical Parameters

Commercial kits were used to measure liver and serum cholesterol, triglycerides, HDL and LDL, as well as the enzymatic activities of alanine aminotransferase (ALT) and aspartate aminotransferase (AST) (Bioclin System II, Belo Horizonte, MG, Brazil). Fasting blood glucose levels were measured using an automated glucose monitor (One Touch Ultra2, Johnson & Johnson Medical S.A., Buenos Aires, Argentina).

### 2.3. Oral Glucose Tolerance Test (OGTT)

An oral glucose tolerance test (OGTT was performed on mice fasted for 6 h. Blood glucose levels were measured at 0, 15, 60, and 120 min following oral glucose overload (2 g/kg), and areas under the glucose curves (AUCs) were determined. Blood glucose levels were measured using an automated glucose monitor (One Touch Ultra2, Johnson & Johnson Medical S.A., Buenos Aires, Argentina). 

### 2.4. Measurement of Hepatic Nitrite (NO_2-_) Concentration

NO production in the liver tissue was quantified by measuring the released nitric oxide (NO) metabolite, nitrite, using Griess reagent (modified, Sigma G4410, St. Louis, MO, USA) according to the manufacturer’s instructions. Nitrite levels were determined using a standard curve prepared against a standard solution of NaNO_2_ [35].

### 2.5. Histopathology

Fragments of the left lateral lobe of the liver were fixed in Millonig’s phosphate-buffered formalin solution (pH 7.2) and embedded in paraffin for light microscopic analysis (Nikon, model 80i and DSRi1 digital camera, Nikon Instruments, Inc., Melville, NY, USA). Histological features of hematoxylin and eosin (H&E)-stained sections were classified into the following categories: Steatosis, inflammation, and hepatocellular damage. Each sample was analyzed using the stereological method of grid-point counting which measures the fatty content of the liver (STEPanizer stereology version 1, Bern, Switzerland) [36,37]. An NAFLD activity score (NAS) was used to classify the stage of liver disease, providing an overall diagnosis for each case as NASH (score ≥ 5), borderline (score 3–4) or not NASH (score 0–2) [38]. Masson’s trichrome stain was used to detect and evaluate fibrosis by collagen quantification using ImageJ (ImageJ version 1.53e, Madison, WI, USA) [39]. For each parameter, five histological sections from six mice per group were analyzed.

### 2.6. Immunohistochemistry

Immunohistochemical analysis of hepatic alpha smooth muscle actin (α-SMA) was performed to determine HSC activation. Briefly, following deparaffinization, sections were treated with 3% hydrogen peroxide (H_2_O_2_) and blocked using non-fat dry milk and bovine serum albumin (BSA). Sections were rinsed in PBS and incubated overnight in a humidified chamber at 4 °C with anti-α-SMA monoclonal primary mouse antibody (Santa Cruz Biotechnology, Santa Cruz, CA, USA) at a dilution of 1:500. The slides were then washed in PBS and treated with a biotinylated secondary antibody, followed by incubation with streptavidin peroxidase, both for 30 min at room temperature. Diaminobenzidine (DAB) was used as the chromogen. Slides were counterstained with Mayer’s hematoxylin. 

### 2.7. RT-PCR

Total RNA was extracted from the middle lobe of the liver using the RNeasy Mini Kit (Qiagen, Düsseldorf, Germany). Next, cDNA synthesis was performed using 1 μg RNA in a final volume of 20 μL via a High Capacity cDNA Reverse Transcription Kit (Applied Biosystems, Beverly, MA, USA). The primers used for product amplification were of liver-type fatty acid-binding protein (*L-FABP*), forward 5′-CCATGACTGGGGAAAAAGTC-3′ and reverse 5′-GCCTTTGAAAGTTGTCACCAT-3′; collagen type I alpha 1 (*COL1A1*), forward 5′-CTCCTGGCAAGAATGGAGAT-3′ and reverse 5′-AATCCACGAGCACCCTGA-3′; and *β-actin* forward 5′-AGATTACTGCTCTGGCTCCTAGC-3′ and reverse 5′-ACTCATCGTACTCCTGCTTGCT-3′. RT-PCR was performed using a Power SYBR Green PCR Master Mix (Applied Biosystems, Beverly, MA, USA) according to the manufacturer’s instructions and run on a 7500 Fast platform (Applied Biosystems, Beverly, MA, USA). The relative expression of the genes of interest was calculated using the ∆∆Ct method and normalized to β-actin expression [17].

### 2.8. Thiobarbituric Acid Reactive Species (TBARs)

Hepatic lipid peroxidation was evaluated by measuring malondialdehyde (MDA) levels using thiobarbituric acid via a spectrophotometric method at an absorbance of 535 nm. The amount of malondialdehyde was estimated as: MDA, ε = 1.56 × 105 M^−1^ cm^−1^ [17,40].

### 2.9. Antioxidant Enzyme Activity

Superoxide dismutase (SOD) activity was measured indirectly via the SOD inhibition index using a spectrophotometer at a wavelength of 530 nm [41]. In order to evaluate catalase (CAT) activity, the consumption of H_2_O_2_ was assessed for 3 min [17] on a SpectraMax M5 ELISA Microplate Reader (Molecular Devices, San Jose, CA, USA) at a wavelength of 230 nm at 30 °C [42].

### 2.10. Intravital Microscopy

For intravital microscopy, mice fasted overnight were anesthetized with ketamine/xylazine (100 mg/kg and 10 mg/kg, respectively, intraperitoneally). After laparotomy, the left liver lobe and epididymal fat were externalized for assessment of microcirculation using intravital microscopy (Olympus BX150WI; Center Valley, PA, USA), as described previously [17]. After intravenously administering 0.3 mg/kg rhodamine 6G (Sigma Chemical Co., St. Louis, MO, USA), leukocyte-endothelial interaction was assessed by counting the number of labeled leukocytes rolling or adhering to hepatic or adipose tissue microcirculation. In the liver, the number of vitamin A-positive hepatic stellate cells (HSCs) was determined as the number of fluorescent cells derived from vitamin A autofluorescence. After intravenously administering 0.05 mL of 2% fluorescein isothiocyanate (FITC)-labeled dextran (molecular weight 150,000; Sigma Chemical Co., St. Louis, MO, USA), hepatic microcirculation images were acquired (Prime Intervision, Doral, FL, USA). Sinusoidal density analysis was performed using ImageJ software (ImageJ 1.47 v; Wayne Rasband, National Institute of Health, Bethesda, MD, USA) to determine the mean value of functional capillary density.

### 2.11. Laser Speckle Flowmetry

Basal microvascular blood flow in liver and epididymal adipose tissues were measured using laser speckle contrast imaging (LSCI) (Pericam PSI system, Perimed, Sweden) as described previously [17] and expressed in arbitrary perfusion units (APUs). Microvascular endothelial reactivity in epididymal adipose tissue was assessed via endothelial response to topical application of the endothelium-dependent vasodilator to acetylcholine (Ach, 10^−8^ mol/m^3^, 10^−7^ mol/m^3^, 10^−6^ mol/m^3^, and 10^−5^ mol/m^3^) (Sigma-Aldrich, St. Louis, MO, USA). Microvascular blood flow responses were expressed as a percentage (%) of deviation from the baseline.

### 2.12. Quantification of Advanced Lipoxidation End Products (ALEs)

Concentrations of fluorescent ALEs in liver tissue were determined as previously described [17]. Briefly, on a SpectraMax M5 ELISA Microplate Reader (Molecular Devices, San Jose, CA, USA), ALEs were measured at a protein concentration of 1 kg/m^3^ at an emission wavelength of 440 nm and an excitation wavelength of 370 nm. A native BSA preparation (1 kg/m^3^, 0.1 mol/m^3^ NaOH) served as reference, and its fluorescence intensity was determined as the unit of fluorescence. The fluorescence values of the samples were expressed in arbitrary units (AUs) [17,43].

### 2.13. Western Blot

Western blotting was used to examine the protein expression of inducible nitric oxide synthase (iNOS) and the receptor for AGE (RAGE). All primary antibodies were purchased from Santa Cruz Biotechnology (Santa Cruz, Santa Cruz, CA, USA) and used at a dilution of 1:500. Liver tissue was homogenized in lysis buffer (20 mmol/m^3^ Trizma/137 mmol/m^3^ NaCl/10% glycerol/1% Nonidet *p*-40/2 mmol/m^3^ EDTA) and centrifuged for 45 min at 14,000× *g* at 4 °C. Protein content was determined via the bicinchoninic acid method using BSA (Sigma-Aldrich, St. Louis, MO, USA) as a standard (BCA Protein Assay Kit, Thermo Scientific, Waltham, MA, USA). Next, 30 μg of protein per lane was separated on a 12% sodium dodecyl sulfate gel and transferred to a polyvinylidene fluoride (PVDF) membrane (Bio-Rad Laboratories, Munich, Germany). After blocking with 5% BSA, the membranes were incubated overnight at 4 °C with primary antibodies. A secondary RDye 680RD donkey-anti-goat antibody (1:15,000; LI-COR Biosciences, Cambridge, UK) for iNOS and a secondary RDye 680RD donkey anti-goat antibody (1:15,000; LI-COR Biosciences, Cambridge, UK) for RAGE were used for detection. The bound complex was detected using the Odyssey Infrared Imaging System (Li-Cor; Lincoln, NE, USA). Images were analyzed using Image Studio Lite software version 4.0.21 (Li-Cor, Lincoln, NE, USA) to obtain integrated intensities and normalized with respect to glyceraldehyde 3-phosphate dehydrogenase (GAPDH) signals (mouse anti-GAPDH monoclonal antibody, 1:30,000; Fitzgerald Industries International, Acton, MA, USA), followed by a secondary IRDye 800CW goat anti-mouse antibody (1:20,000; LI-COR Biosciences).

### 2.14. Statistical Analysis

Results are expressed as the mean ± SEM of each group. Normality was assessed using the Shapiro–Wilk test, and comparisons between groups were made using two-way analysis of variance (ANOVA) followed by Tukey’s post hoc test. The relationship between ALEs and metabolic and microcirculatory parameters was examined using Pearson’s correlation coefficient and linear regression. Data were analyzed using GraphPad Prism 8.0.1 (GraphPad Software Inc., La Jolla, CA, USA) and R software (version 3.4.2; R Core Team, R Foundation for Statistical Computing, Vienna, Austria). Statistical significance was set at *p* < 0.05.

## 3. Results

### 3.1. SV Protects against NAFLD-Induced Metabolic Changes

**Figure 2:**
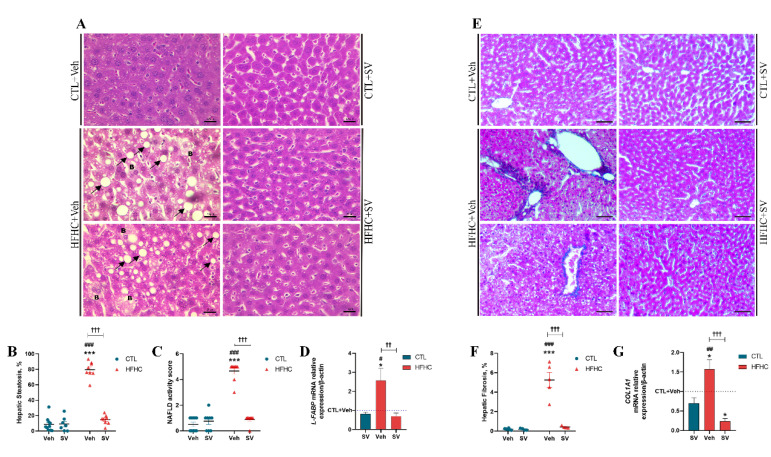
SV protects against HFHC-induced hepatic steatosis and fibrosis progression. Representative images of histological analyses via hematoxylin-eosin staining of the livers from mice fed control diet (CTL+Veh), control diet plus SV (CTL+SV), HFHC diet (HFHC+Veh), or HFHC diet plus SV treatment (HFHC+SV) (**A**). Quantification of percentage of hepatic steatosis is represented in (**B**) and NAFLD activity score (NAS) in (**C**). Real-time PCR analyses of mRNA transcript levels of gene encoding liver-type fatty acid-binding protein (*L-FABP*) is shown in (**D**). Representative images of histological analyses via Masson’s trichrome staining of the livers from the CTL+Veh, CTL+SV, HFHC+Veh or HFHC+SV group (**E**). (**F**) Demonstration of quantification of percentage of hepatic fibrosis area. Real-time PCR analyses of mRNA transcript levels of gene encoding collagen type I alpha 1 (*COL1A1*) is shown in (**G**). Arrow: Lipid vacuoles; Letter B: Hydropic degeneration. * *p* < 0 vs. CTL+Veh; *** *p* < 0.001 vs. CTL+Veh; ^#^ *p* < 0.05 CTL+SV; ^##^ *p* < 0.01 CTL+SV; ^###^ *p* < 0.001 vs. CTL+SV; ^††^ *p* < 0.01 HFHC+SV; ^†††^ *p* < 0.001 HFHC+SV.

To explore the effects of SV on fatty liver disease, mice were fed either control or HFHC purified diets and subgroups of control and HFHC-fed mice were treated with SV (Figure 1A). All parameters were similar in the groups of mice fed with normal chow. Both HFHC-fed and HFHC+SV mice gained more weight than the controls fed normal chow (*p* < 0.001); (Figure 1B). HFHC-fed as well as HFHC+SV mice displayed impaired glucose tolerance (*p* < 0.05) compared to the controls (Figure 1C). HFHC-fed and HFHC+SV mice showed significantly higher fat depots compared to those of the controls (Figure 1D). All groups had similar liver weights and liver/body weight ratios (Figure 1E). Furthermore, HFHC-fed mice showed significantly increased hepatic cholesterol and triglycerides, serum cholesterol (total and LDL), and triglycerides, as well as ALT and AST enzyme activity and a reduction in HDL cholesterol, compared to those of control mice (Figure 1F). Histologically, steatosis (Figure 2A,B), hepatocyte ballooning (Figure 2A), and fibrosis (Figure 2E,F) were observed in the livers of HFHC-fed mice, while no inflammatory cell infiltration was observed (Figure 2A). Accordingly, steatosis, hepatocyte ballooning, and fibrosis were significantly higher in HFHC-fed vs. control and HFHC+SV mice livers, contributing to significantly higher NAFLD activity scores in HFHC-fed mice livers (all *p* < 0.001; Figure 2C). HFHC-fed mice showed increased mRNA transcripts of L-FABP in the liver (Figure 2D). 

### 3.2. SV Prevents Microcirculatory Dysfunction in HFHC-Fed Mice

**Figure 3:**
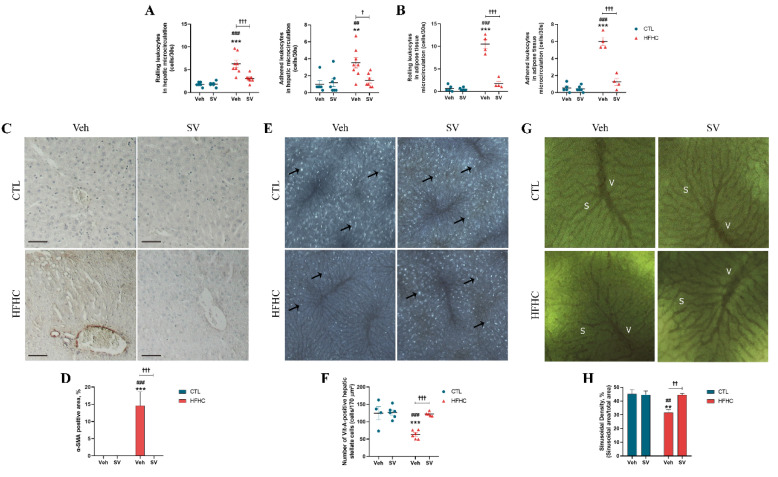
SV prevents microcirculatory dysfunction in HFHC-fed mice. Liver (**A**) and adipose tissue (**B**) microcirculation assessed via intravital microscopy following intravenous administering of rhodamine 6G with quantification of adhesion and rolling of leukocytes. Representative immunohistochemical staining of liver sections showing expression of alpha smooth muscle actin (α-SMA) in mice fed with control diet (CTL+Veh), control diet plus SV (CTL+SV), HFHC diet (HFHC+Veh), or HFHC plus SV treatment (HFHC+SV) (**C**). Quantification of the percentage of α-SMA-positive area is represented in (**D**). In vivo distribution (**E**) and quantification (**F**) of vitamin A-positive cells indicative of retinoid storage in cytoplasmic droplets of hepatic stellate cells. Representative intravital fluorescence microscopic images (**G**) and quantification (**H**) of sinusoidal perfusion density in the liver following intravenous administering of fluorescein isothiocyanate-labeled dextran. ** *p* < 0.01 vs. CTL+Veh; *** *p* < 0.001 vs. CTL+Veh; ^##^ *p* < 0.01 CTL+SV; ^###^ *p* < 0.001 vs. CTL+SV; ^†^ *p* < 0.05 HFHC+SV; ^††^ *p* < 0.01 HFHC+SV; ^†††^ *p* < 0.001 HFHC+SV.

The effects of SV on microcirculatory parameters were assessed using intravital microscopy and LSCI flowmetry. HFHC-fed mice displayed significantly higher numbers of rolling and adherent leukocytes in hepatic and adipose microcirculation than the controls fed with normal chow, but this was not observed in HFHC+SV-fed mice (Figure 3A,B). HSC activation in HFHC-fed mice was increased compared to that of control mice fed with normal chow (both *p* < 0.001), as evidenced by increased α-SMA protein expression in the liver tissue (Figure 3C,D) and decreased vitamin A-positive cell numbers (*p* < 0.001) (Figure 3E,F); however, such an increase was not observed in HFHC+SV mice. Accordingly, HFHC-fed mice showed increased mRNA transcripts of collagen 1α compared to control and HFHC-treated mice, as indicated by qPCR analysis (Figure 2G). Furthermore, the hepatic capillary network architecture as well as its vascular density was altered in HFHC-fed mice, as indicated by a 30% reduction in sinusoid density and distorted sinusoidal network, compared to those of control and HFHC+SV mice (Figure 3G,H).

**Figure 4:**
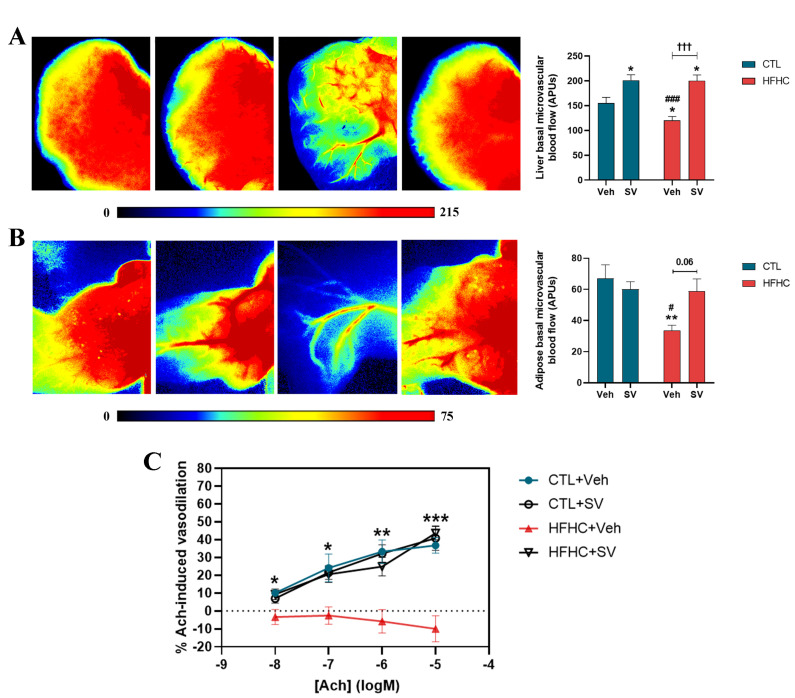
Effect of SV on hepatic and visceral adipose tissue microvascular perfusion. Liver and epididymal adipose tissue microvascular blood flow assessed by laser speckle contrast imaging (LSCI). Representative images of LSCI and microvascular blood flow quantification in the left lateral hepatic lobe (LLL) (**A**) or epididymal fat deposit (**B**) in mice fed with control diet (CTL+Veh), control diet plus SV (CTL+SV), HFHC diet (HFHC+Veh), or HFHC diet plus SV treatment (HFHC+SV). The color scale represents blood flow in arbitrary perfusion units (APUs). Percentage of change in adipose tissue blood flow following topical administering of crescent doses of 2% acetylcholine (Ach) is demonstrated in (**C**). * *p* < 0.05 vs. CTL+Veh; ** *p* < 0.01 vs. CTL+Veh; *** *p* < 0.001 vs. CTL+Veh; ^#^ *p* < 0.05 CTL+SV; ^###^ *p* < 0.001 vs. CTL+SV; ^†††^ *p* < 0.001 vs. HFHC+SV.

Basal hepatic and adipose tissue microvascular blood flow was significantly decreased in HFHC-fed mice compared with that of the control or HFHC+SV groups (Figure 4A,B). Response of the adipose tissue endothelium-dependent vasodilator to Ach in HFHC mice was impaired in a dose-dependent manner, compared to that in normal chow-fed or HFHC-fed mice (Figure 4C).

### 3.3. SV Protects against NAFLD-Induced Oxidative Damage and Inflammation

**Figure 5:**
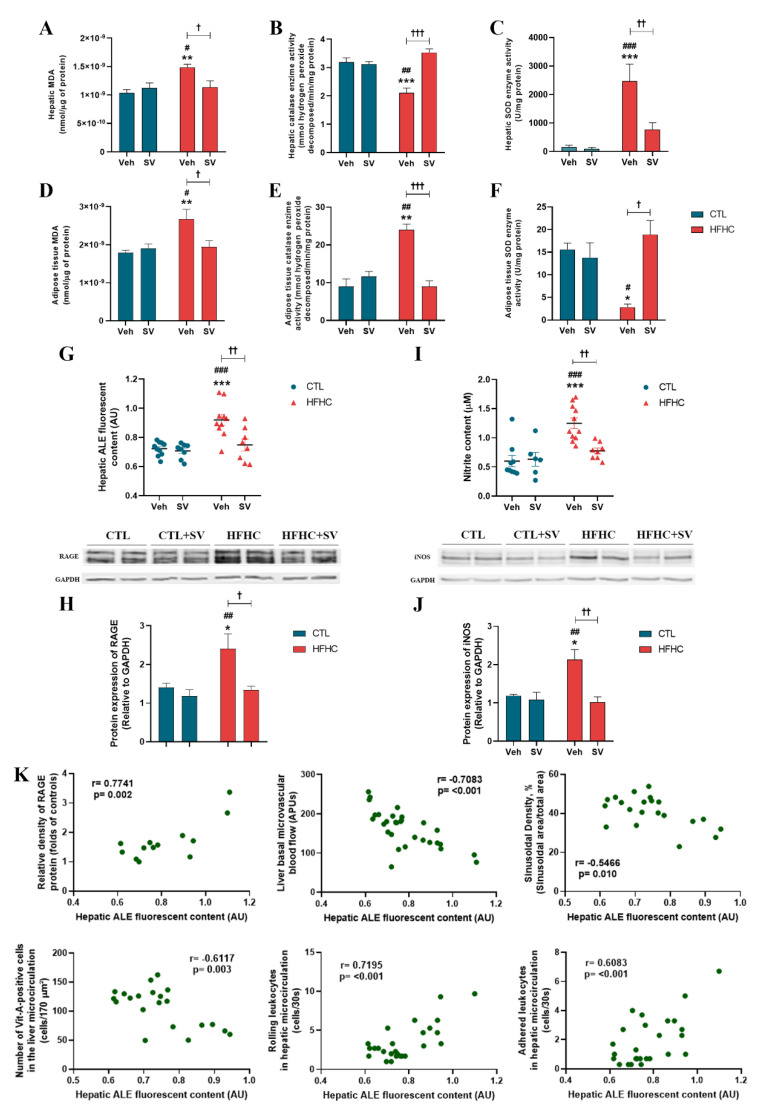
SV protects against NAFLD-induced oxidative damage and inflammation. Levels of malondialdehyde (MDA) indicating lipid peroxidation assessed by thiobarbituric acid reactive species (TBARs) (**A**,**D**), catalase enzyme activity (**B**,**E**) and superoxide dismutase enzyme activity (**C**,**F**) in the liver and epidydimal adipose tissues in mice fed with control diet (CTL+Veh), control diet plus SV (CTL+SV), HFHC diet (HFHC+Veh), or HFHC diet plus SV treatment (HFHC+SV). Hepatic advanced lipoxidation end product (ALE) deposition (**G**), receptors of advanced glycation end products (RAGE) protein expression in the liver (**H**), nitrite levels indicative of NO (**I**), and iNOS protein expression (**J**). Pearson’s correlation analysis between ALE deposition in the liver and metabolic and microcirculatory parameters (**K**). * *p* < 0.05 vs. CTL+Veh; ** *p* < 0.01 vs. CTL+Veh; *** *p* < 0.001 vs. CTL+Veh; ^#^ *p* < 0.05 vs. CTL+SV; ^##^ *p* < 0.01 vs. CTL+SV; ^###^ *p* < 0.001 vs. CTL+SV; ^†^ *p* < 0.05 vs. HFHC+SV; ^††^ *p* < 0.01 vs. HFHC+SV; ^†††^ *p* < 0.001 vs. HFHC+SV.

Examination of hepatic and adipose tissue oxidative stress via TBARs revealed that lipid peroxidation in HFHC-fed mice was significantly increased compared to that in the control or the HFHC+SV group, (Figure 5A,D). The activity of the antioxidant catalase (CAT) was significantly decreased (Figure 5B), while that of superoxide dismutase (SOD) was increased (Figure 5C) in the liver of HFHC-fed mice, compared to that of the control or HFHC-treated group. The adipose tissue of HFHC mice showed increased CAT activity (Figure 5E) and decreased SOD activity (Figure 5F) compared to that of control mice fed with normal chow and HFHC+SV. ALE deposition in the liver tissues of HFHC-fed mice was increased, but such an increase was not observed in normal chow-fed mice or HFHC+SV mice (Figure 5G). Similarly, RAGE protein expression was significantly increased in HFHC-fed mice compared to that in the control and HFHC+SV groups (Figure 5H). Furthermore, increases in hepatic nitric oxide bioavailability (Figure 5I) and iNOS protein expression (Figure 5J) were observed in mice fed with HFHC diet compared to those fed with normal chow or HFHC+SV mice.

Hepatic ALE deposition correlated with RAGE and microcirculatory parameters. A strong positive correlation was observed between hepatic ALE levels and hepatic RAGE protein expression levels and hepatic rolling of leukocytes. The analysis also demonstrated a moderate correlation with hepatic adhesion of leukocytes (Figure 5K). Pearson’s correlation coefficient showed a strong negative correlation between hepatic ALE deposition and hepatic basal microvascular blood flow and a moderate negative correlation with the number of vitamin A-positive HSCs and sinusoidal perfusion density in the liver, indicating that the increase in these variables is inversely proportional to the increase in hepatic ALE (Figure 5E,K).

## 4. Discussion

While the protective effects exerted by SV against NAFLD are documented [23,31,44], information regarding the mechanisms underlying such protection is scant. In this study, we demonstrate, for the first time, that SV protects against high-fat–high-carbohydrate diet-induced hepatic and adipose tissue microcirculatory dysfunction. Furthermore, we show that amelioration of microcirculatory disturbances was associated with decreased lipid peroxidation and ALE-RAGE axis activation, suggesting that SV contributes to microcirculatory recovery by modulating oxidative and ALE-RAGE stress.

As previously evidenced during pre-clinical and clinical studies, SV improves serum and hepatic lipid profiles and liver transaminases, thus impairing steatosis and NAFLD progression [45,46,47,48,49,50,51]. Management of dyslipidemia, liver function and steatosis is considered crucial for attenuating the progression of NAFLD [52]. Furthermore, SV decreased *L-FABP* expression. L-FABP mRNA is highly expressed in HFHC-fed mice, and such increased expression is associated with steatosis, as L-FABP plays a role in lipid and fatty acid metabolism [53]. L-FABP knockout protects Western diet-fed mice from developing hepatic steatosis and fibrogenesis [54,55,56]. Previously, Landrier et al. showed that SV treatment induced a dose-dependent increase in L-FABP mRNA levels in isolated rat hepatocytes and in vivo [57]. Regarding their in vitro results, the complex intracellular cross-talk and lipid signalling that occurs in vivo between hepatocytes and other cells such as HSCs should not be neglected, underscoring the importance of in vivo models for understanding complex systems [56]. Regarding the in vivo results, Landrier and colleagues used only three rats/group and a statin dose of 100 mg/kg for five consecutive days, which is a very high and unusual dose and a very short treatment duration. In addition, the differences in the protocol between Landrier and colleagues and ours make comparative analysis difficult. In another study, Song et al. showed that there was no significant upregulation of lipid transporter genes such as CD36 and FABPs in hepatocytes treated with lovastatin or SV [58]. Therefore, the effects of statins on genes involved in lipid metabolism are still controversial and require further studies.

A major objective of this study was to investigate the pathophysiological processes that occur in the microcirculation of mice with NAFLD, as well as the potential of simvastatin to modulate these processes. The unique anatomical relationship between the liver and adipose tissue, which are connected by the portal circulation, indicates that studying the hepatic and visceral adipose tissue microcirculation is clinically relevant. Thus, we utilized in vivo imaging techniques to analyze the microcirculation of epididymal fat deposits, as being representative of visceral adipose tissue (VAT), as well as hepatic microcirculation. Consistent with previous studies, we showed that the liver of mice with NASH undergo (a) a substantial decrease in capillary density, (b) decreased basal blood flow in the hepatic microcirculation, (c) a pronounced increase in HSC activation, and (d) increased leukocyte recruitment in the sinusoidal and post-sinusoidal venules [17,20,59]. Furthermore, SV rescued liver microcirculation alterations induced by NAFLD. Decreased lipid accumulation and steatosis induced by SV may reduce the narrowing of sinusoids in the hepatocytes of NAFLD mice and thus promote blood flow recovery in microcirculation [17,18,60]. Moreover, as activated HSCs play a pivotal role in collagen deposition as well as in vascular tone regulation [61,62], the protective effect exerted by SV against HSC activation may significantly impact fatty liver progression [45]. Our analysis showed decreased fibrosis and positive α-SMA staining, in addition to a reduction in collagen I gene expression in the livers of HFHC-fed mice treated with SV. Substantiating this, Gracia-Sancho et al. demonstrated that SV maintains HSCs in a quiescent state in a cirrhotic liver and thereby significantly reduces liver fibrosis [63]. Bravo and coworkers demonstrated in sorted cells from rats with NASH that the vasoprotective effect of statins was associated with regression of HSC activation and restoration of LSEC differentiation [64]. In addition, accumulations of circulating leukocytes, that are mechanically trapped in or adhered to the vasculature, may further exacerbate sinusoid compression [20]. Thus, SV may protect the microcirculation by decreasing leukocyte recruitment [65]. SV improved vascular disturbances and ameliorated portal hypertension in carbon tetrachloride-induced cirrhotic rats, by increasing nitric oxide bioavailability and improving liver sinusoidal endothelial function [66]. In cirrhotic livers, SV enhanced KLF2-dependent vasoprotective mechanisms, thereby preventing liver damage, inflammation and oxidative stress and improving endothelial dysfunction [67]. This was also observed in explanted rat livers when SV was added to the storage solution [68]. The observed reversal of functional capillary density in the hepatic microcirculation by SV may be a consequence of the decrease in (i) steatosis, (ii) fibrosis, (iii) HSC activation and *COL1A1* expression and (iv) leukocyte recruitment, which in turn reversed tissue hypoxia and recovered microvascular blood flow. 

Our analysis of VAT microcirculation revealed that mice fed with an HFHC diet exhibited endothelial dysfunction, as evidenced by an impaired vasodilatation response to Ach. Similarly, mice with NAFLD showed reduced blood perfusion and increased leukocyte recruitment in the VAT microvasculature. While studies have demonstrated leukocyte infiltration of adipose tissue in obese mice and humans [69,70], to our knowledge, ours is the first study to evaluate VAT vascular beds in NAFLD. As body weight increases, adipose tissue expands via hypertrophy or hyperplasia (increased adipocyte size or increased adipocyte number, respectively). However, hypertrophy is not accompanied by an increase in capillary number, and may result in adipose tissue hypoxia, tissue remodeling, leukocyte recruitment, and tissue inflammation [71,72,73,74]. Crosstalk between adipose tissue and the liver may further exacerbate the pathophysiological processes associated with NAFLD [75]. Adipose tissue hypertrophy and hyperplasia increase the release of non-esterified free fatty acids (FFAs) and proinflammatory cytokines into the circulation, resulting in ectopic lipid deposition, low-grade chronic inflammation, and insulin resistance [76]. Concomitantly, adipose tissue–derived factors may be released into the circulation and target the liver, thereby aggravating liver injury [77,78] and subsequently impairing microcirculation function. Notably, microcirculation dysfunction in the VAT was fully recovered by SV.

In order to elucidate the mechanisms by which SV protects against hepatic and VAT microcirculatory dysfunction, we analyzed alterations in the balance between pro-oxidant and antioxidant factors in these tissues due to lipid peroxidation, antioxidant enzyme activities, and the ALE-RAGE axis. We further observed that an increase in hepatic and VAT MDA levels, which caused an increase in lipid oxidation in NAFLD-induced mice, acted as a key insult contributor to the progression of NAFLD. Concomitantly, the first line of the antioxidant defense mechanism, SOD and CAT, which protects the organism from ROS-mediated oxidative damage, were altered in the liver and VAT of mice with NAFLD, as was previously reported [79,80,81]. Increased antioxidant enzyme activity may indicate an adaptive response that counteracts an increase in lipid peroxidation and ROS in NAFLD because increased oxidative stress in the tissues causes cell damage, induces cytokine production, and activates inflammatory cells, which further stimulates ROS formation [82,83]. As NAFLD progresses, antioxidant reserves may be depleted, resulting in a lower antioxidant capacity. Previous studies have demonstrated that increased oxidative stress pathway activation is associated with reduced microvascular blood flow [17,42,84,85]. Notably, we showed that SV restored the oxidative balance in mice fed with an HFHC diet. Statins reduce the production of superoxide anions via NADPH oxidase [86] by directly affecting this enzyme complex [87] which leads to the inhibition of Rac1 prenylation [88]. Inhibition of protein prenylation by SV results in the modulation of inflammatory and oxidative responses [89]. In addition, SV activates antioxidant enzymes via Nrf2, a key regulator of the antioxidant defense machinery [44,90].

We found that SV therapy reversed a significant increase in ALE deposition and RAGE protein expression in hepatic tissues. Lipid peroxidation, induced by oxidative stress, causes an increase in the endogenous production of reactive carbonyl species (RCS) and their derivatives, such as MDA, giving rise to ALEs such as MDA-Lys, HNE-Lys, FDP-Lys, carboxymethyl-lysine (CML), and S-carboxymethyl-cysteine, among others [91]. ALEs regulate a multitude of cell signaling pathways and play a role in a wide range of molecular and biological processes, ranging from protein, DNA, and phospholipid damage to signaling pathway activation and/or modification in response to oxidative stress stimuli [92,93]. ALEs exert their pathogenic effects by binding to receptors of advanced glycation end products (AGEs), termed RAGEs, which increases inflammatory, oxidative, and fibrotic responses [94]. Endothelial cells, vascular smooth muscle cells, peripheral blood mononuclear cells, macrophages (including Kupffer cells), and HSCs are among the cells that express ALE receptors [95]. AGEs and ALEs are formed by Maillard reactions and share the same receptor, thereby eliciting similar responses. Activities associated with the ALE/AGE-RAGE pathway are reportedly increased in liver diseases [96,97,98,99,100]. Leung et al. demonstrated a higher deposition of CML in the liver of animals with NAFLD, induced by a diet deficient in methionine and choline, and an increase in the production of ROS by Kupffer cells dependent on the AGE-RAGE pathway [101]. Furthermore, a high intake of ALEs/AGEs has been shown to increase hepatic CML contents, thereby worsening liver damage and increasing levels of proinflammatory cytokines by activating HSCs in a RAGE pathway-dependent manner, which in turn leads to increased oxidative stress and fibrosis [102,103]. Increased oxidative stress and tissue inflammation caused by ALEs support the “multiple-hit hypothesis,” suggesting that ALEs play a key role in the progression of simple steatosis to NASH and liver fibrosis. On the other hand, studies have shown that statins may act as negative regulators of RAGE and reduce ALEs [17], in addition to inducing an increase in sRAGE, a soluble form of RAGE that can act as an AGE/ALE scavenger [17]. Patients with hypercholesterolemia had lower levels of sRAGE than patients with normocholesterolemia, while plasma sRAGE levels in patients with statin-treated hypercholesterolemia were significantly higher than those in untreated individuals. In addition, sRAGE levels were inversely correlated with LDL levels (r = 0.36, *p* = 0.005) and total cholesterol levels (r = 0.326, *p* = 0.011) [104]. A study on type 2 diabetes demonstrated that atorvastatin reduced serum CML levels in hypercholesterolemic patients without any cardiovascular diseases. It was reported that blood glucose control levels were not altered by treatment with atorvastatin, and changes in CML were not correlated with those in lipid parameters [105], suggesting that statins may lower serum CML levels in a manner independent of its lowering of cholesterol.

Free radical nitric oxide (NO) plays an important role in the liver physiology and pathophysiology of NAFLD [106]. Many studies have discussed the vasoprotective role of NO generated by endothelial nitric oxide synthase (eNOS) in NAFLD; however, NO activity carries advantages as well as disadvantages. In the liver, small amounts of NO generated by eNOS are believed to promote liver homeostasis and protect against pathological conditions. By contrast, iNOS expression, which is absent in resting cells, is induced by immune stimuli and inflammatory cytokines during inflammation and plays a role in the development and maintenance of many liver diseases [106,107]. Moreover, proinflammatory cytokines, such as IL−1 and TNF-α, may induce iNOS expression in liver sinusoidal endothelial cells and hepatocytes [108,109,110]. NO is produced by activated HSCs as well, due to the action of iNOS [62,111,112]. Our findings confirmed that iNOS is overexpressed in the liver tissues of animals with NAFLD, together with an important increase in the NO metabolite, nitrite. Increased iNOS expression generates oxidative stress and leads to a proinflammatory condition that affects liver microcirculation [113,114]. SV treatment reduced iNOS expression and NO production. Statins decreased the activity of activating protein-1 (AP−1), a transcription factor that plays an important role in endothelial cell inflammatory responses and regulates genes responsible for cytokines, adhesion molecules, and iNOS production [115]. Statins negatively regulate the expression of iNOS in the endothelium and small kidney vessels of transgenic mice that express human renin and angiotensinogen [116], leading to increased eNOS activity and NO bioavailability, thereby preventing an increase in iNOS in rats after ischemia-reperfusion [117]. Furthermore, SV decreased iNOs and NO through decreased HSC activation [45].

**Figure 6:**
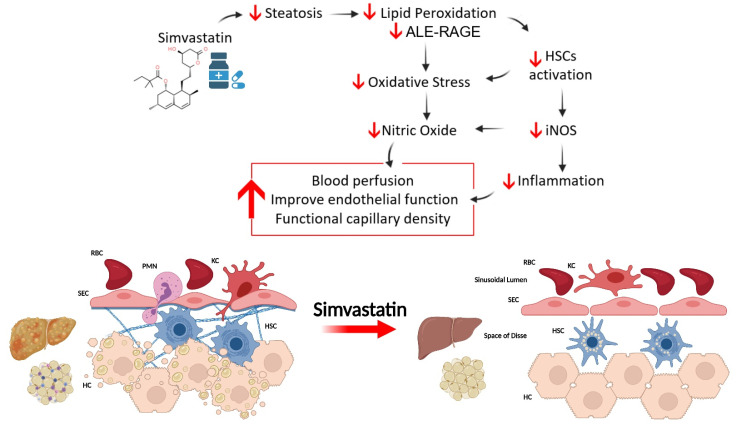
The impact of oral treatment with SV on the development of NAFLD. SV acts on multiple key insults that together contribute to the onset and progression of NAFLD. In this context, SV protects against NAFLD-induced (i) metabolic changes, (ii) microcirculatory dysfunction, and (iii) oxidative damage and inflammation. HSC: Hepatic stellate cells; RBC: Red blood cell; PMN: Polymorphonuclear leukocytes; KC: Kupffer cells; SEC: Liver sinusoidal endothelial cells; HC: Hepatocytes.

Pearson’s correlation analyses using NAFLD progression factors demonstrated that hepatic ALE levels were correlated with the modulation of key metabolic and microcirculatory parameters, including RAGE hepatic expression, liver basal microvascular blood flow, sinusoidal density, HSC activation, and leukocyte recruitment, which strengthens the hypothesis that advanced lipoxidation end products are strongly related to the pathophysiology of NAFLD and microcirculatory changes (Figure 6), in addition to being an important factor in the modulation of NAFLD complications.

## 5. Conclusions

Our findings suggest that SV exerts pleiotropic effects, associated with negative modulation of lipid accumulation and peroxidation, ALE-RAGE pathway, oxidative stress, and iNOS expression, protects microcirculatory dysfunction associated with NAFLD, and recovers tissue perfusion, thereby contributing to the repositioning of statins for NAFLD treatment.

## Data Availability

Data related to this study are contained within the article.
